# How to Have Good Teeth

**Published:** 1877-12

**Authors:** A. Homer Trego


### *How to Have Good Teeth.*

BY A. HOMER TREGO, D. D. S.

There is scarcely a subject of a personal character so sadly neglected and so little understood, by the people in general, as the care of the teeth.

In view of this fact, in 1869, the Odontographic Society, of Philadelphia (about 100 members of eminent ability) offered a prize for the best essay on the subject; the same to be published for the benefit of the public.

The prize was awarded for the following:

#### RULES FOR PRESERVING THE TEETH.

1st. *Cleanse* your teeth once, or oftener, every day! *Always* cleanse them before retiring at night! *Always* pick the teeth and rinse the mouth after eating!

2d. *Cleansing the teeth* consists in thoroughly removing every particle of foreign substance from around the teeth and gums.

3d. *To cleanse*, use well made brushes, soft quill or wood toothpicks, an *antacid, styptic toothwash and precipitated chalk.* If these means fail apply to a reliable dentist.

4th. *Always* roll the brush up and down lengthwise of the teeth, by which means you may avoid injuring the gums and necks of the teeth, and more thoroughly cleanse between them.

5th. *Never* use a dentifrice containing acid, alkali, charcoal, soap, salt, or any gritty or powerful detersive substance.

6th. *Powders* and *pastes* generally are objectionable. They injure the gums and soft parts of the teeth, and greatly assist in forming tartar. *A wash*, properly medicated and carefully prepared, is pleasanter and more beneficial. It dissolves the injurious secretions and deposits, and the whole is readily removed with the brush and water.

7th. *Avoid eating hot food!* Thoroughly masticate and insalivate the food before swallowing it. Frequent indulgence in sweetmeats, etc., between regular meals, disturbs the regular process of digestion, and a viscid secretion is deposited in the mouth (from the stomach,) which is very injurious to the teeth.

8th. *Parents!* Carefully attend to your children's second dentition. Gently prevail upon them, at an early age, to visit, at frequent intervals, a careful and skillful operator.

Remember that four of the permanent double teeth came in at about the age of six years. They are very liable to decay early, are very large, and should never be allowed to require extracting.

Children do not " shed " their teeth as they did in former ages. Instead of being trained to masticate nutritious food they are tempted with and allowed to " gulp down " delicacies, hot cakes, hot beverages, etc. Thus, by depriving the teeth of their natural function and overtasking the stomach, a morbid condition of the general system is produced; the " first teeth " are prematurely decayed, and the permanent set are not matured at the proper period of dentition. The consequences are terrible.

9th. *Never allow any one to extract a tooth*, or to dissuade you from having them filled, unless absolutely necessary. Many so-called dentists, actuated by selfish motives advise extracting, and sacrifice many teeth which competent operators can render serviceable for many years.

10th. *Carelessness and procrastination* are responsible for a large majority of teeth that are lost.— *Western Science Review.*

